# Exploring the Extent of the Hikikomori Phenomenon on Twitter: Mixed Methods Study of Western Language Tweets

**DOI:** 10.2196/14167

**Published:** 2019-05-29

**Authors:** Victor Pereira-Sanchez, Miguel Angel Alvarez-Mon, Angel Asunsolo del Barco, Melchor Alvarez-Mon, Alan Teo

**Affiliations:** 1 Department of Psychiatry Clinica Universidad de Navarra Pamplona Spain; 2 Department of Surgery, Medical and Social Sciences University of Alcala Madrid Spain; 3 Instituto Ramon y Cajal de Investigaciones Sanitarias Madrid Spain; 4 Department of Epidemiology & Biostatistics Graduate School of Public Health and Health Policy University of New York New York, NY United States; 5 Department of Medicine and Medical Specialities University of Alcala Madrid Spain; 6 Service of Internal Medicine, Autoimmune Diseases and Rheumatology Hospital Universitario Principe de Asturias Madrid Spain; 7 Department of Psychiatry Oregon Health & Science University Portland, OR United States; 8 School of Public Health Oregon Health & Science University and Portland State University Portland, OR United States; 9 Center to Improve Veteran Involvement in Care, VA Portland Health Care System, Department of Veterans Affairs (VA) Portland, OR United States

**Keywords:** social isolation, loneliness, hikikomori, hidden youth, social media, Twitter, social withdrawal

## Abstract

**Background:**

Hikikomori is a severe form of social withdrawal, originally described in Japan but recently reported in other countries. Debate exists as to what extent hikikomori is viewed as a problem outside of the Japanese context.

**Objective:**

We aimed to explore perceptions about hikikomori outside Japan by analyzing Western language content from the popular social media platform, Twitter.

**Methods:**

We conducted a mixed methods analysis of all publicly available tweets using the hashtag #hikikomori between February 1 and August 16, 2018, in 5 Western languages (Catalan, English, French, Italian, and Spanish). Tweets were first classified as to whether they described hikikomori as a problem or a nonproblematic phenomenon. Tweets regarding hikikomori as a problem were then subclassified in terms of the type of problem (medical, social, or anecdotal) they referred to, and we marked if they referenced scientific publications or the presence of hikikomori in countries other than Japan. We also examined measures of interest in content related to hikikomori, including retweets, likes, and associated hashtags.

**Results:**

A total of 1042 tweets used #hikikomori, and 656 (62.3%) were included in the content analysis. Most of the included tweets were written in English (44.20%) and Italian (34.16%), and a majority (56.70%) discussed hikikomori as a problem. Tweets referencing scientific publications (3.96%) and hikikomori as present in countries other than Japan (13.57%) were less common. Tweets mentioning hikikomori outside Japan were statistically more likely to be retweeted (*P*=.01) and liked (*P*=.01) than those not mentioning it, whereas tweets with explicit scientific references were statistically more retweeted (*P*=.01) but not liked (*P*=.10) than those without that reference. Retweet and like figures were not statistically significantly different among other categories and subcategories. The most associated hashtags included references to Japan, mental health, and the youth.

**Conclusions:**

Hikikomori is a repeated word in non-Japanese Western languages on Twitter, suggesting the presence of hikikomori in countries outside Japan. Most tweets treat hikikomori as a problem, but the ways they post about it are highly heterogeneous.

## Introduction

*Hikikomori* is the romanization of a Japanese concept referring to a complex phenomenon characterized by severe social withdrawal [1-3]. Definitions of hikikomori have evolved slightly over time [4], but in general, individuals with hikikomori are defined by their pattern of social isolation, remaining usually at their homes, which includes significant distress or functional impairment (eg, inability to maintain academic studies or a job) and a duration of at least 6 months [5-7].

This concept was developed in the last decades of the 20th century in Japan [2], where it is widely recognized as a heterogeneous psychiatric condition, with an estimated prevalence greater than 1% among adults in that country [8]. The extent of this condition has become a major source of concern among many health professionals and policymakers in Japan, as it usually affects young individuals vulnerable to psychological distress, social stigma or cultural marginalization [9], worse physical health [10], and loss of opportunities for education and work. Consequently, hikikomori implicates suffering for not only the affected individual but also his or her family [11], and, at a large scale, it jeopardizes the labor market and public health [12].

In recent years, clinicians and researchers across the world have reported the existence of patients with similar patterns of severe social withdrawal [13], including in Hong Kong [14], mainland China [15], South Korea, India and the United States [6], Oman [16], Spain [17-20], Italy [21], France [22,23], and Brazil [24]. Recently, researchers in Spain characterized 190 patients with social withdrawal meeting the definition of hikikomori, constituting one of the largest described such cohorts outside of Japan [17]. Together, these observations have challenged the view of hikikomori as a syndrome restricted to Japan, a revelation that was hinted at years ago [4]. These studies also raise questions as to whether the extent of hikikomori varies across countries and points in time.

Despite the growing observation of hikikomori globally, there has been an alternative viewpoint that hikikomori does not represent a form of psychopathology; rather, it should be considered a *nonproblematic* self-imposed *lifestyle* of isolation [25]. This consideration of hikikomori frequently falls in the discussion of the so-called *not in employment, education nor training* (NEET) individuals [9]. Accordingly, some authors have suggested that hikikomori might be a reaction to the pressures of a globalized and postindustrialized Japanese society from individuals who consciously refuse to adopt the mainstream cultural values, perhaps a *social pathology* rather than a psychiatric one [25,26].

Individuals with *hikikomori* are usually a hard-to-reach population owing to the fact that hikikomori's defining feature (social isolation) prevents or delays the presentation of these individuals to clinical care and their involvement in research. Some have hypothesized that hikikomori and internet addiction are closely correlated, under the presumption that individuals with hikikomori spend much of their time retreating to anonymous and impersonal Web spaces [27]. Accordingly, the internet, and particularly social media, may be a unique space where these individuals might seek peer help and where they could be reached, identified, and supported by mental health professionals.

Twitter is one of the most popular social media platforms in Western countries and allows users to publicly share and interact with short posts (tweets) [28]. This environment has been the focus of an increasing amount of quantitative and qualitative medical research, using a wide range of different approaches, from descriptive metrics of tweets to manual or machine-learning content analysis regarding disorders such as Alzheimer’s disease [29], epilepsy [30], breast cancer [31], anorexia nervosa [32], schizophrenia [33], and depression [34]. Twitter is a unique venue to study the feelings, beliefs, knowledge, and behaviors of large numbers of people, particularly young ones, and has been proposed as a great source of infodemiologic data to survey, track, and predict medical problems [35-38]. In addition, previous research from our team has highlighted the increasing interest among Twitter users in psychiatric disorders [28]. Thus, Twitter constitutes a rich social context and online community to explore mental disorders and to identify and reach otherwise hard-to-reach individuals [39,40]. Using Twitter in the study of hikikomori offers several potential strengths. Aspects such as language of tweets (as a proxy of users’ geographical and cultural background) and time trends can be examined. In addition, content analysis could be used to classify tweets into different topics or categories of interest. Finally, analyzing Twitter content related to hikikomori could inform the development of future social media–based interventions for individuals with hikikomori to provide users with accurate information, fight stigma, and reach a target population, which might be unlikely to leave their rooms to ask for help by themselves. These kinds of interventions, using other social media platforms, have started to prove effective in people with hikikomori [27] and other potentially margined populations such as the military veterans [41].

The primary aims of this study are to (1) describe and categorize the content of tweets regarding hikikomori in several Western languages; (2) identify what content related to hikikomori generates the most interest (retweets, likes, and associated hashtags); and (3) explore temporal trends in hikikomori on Twitter.

## Methods

### Study Design and Data Source

This study was designed as a mixed methods analysis of quantitative Twitter metrics and qualitative content from recent publicly available tweets about hikikomori in languages used in Western countries where hikikomori has been described. The inclusion criteria for tweets were (1) being public (nonprivate); (2) use of the hashtag #hikikomori; (3) posted between February 1 and August 16, 2018; and (4) text in English, Italian, Spanish, Catalan, or French. The exclusion criteria were (1) no identifiable language or (2) only contained a link (ie, spam tweets).

Twitter provides 3 primary sources of data: Twitter’s Search application programming interface (API), Twitter’s Streaming API, and Twitter’s Firehose. Twitter’s Firehose is the only one that has access to 100% of Twitter content. Twitter’s Firehose formerly was handled by multiple data providers (eg, Gnip, DataSift, and Topsy), although, since August 2015, Twitter only allows access to Twitter’s Firehose through Gnip [28,42]. Tweet Binder, the search engine we employed, uses Twitter Firehose [43] and allows access to 100% of all public tweets that match a set of search criteria (query) [44]. In addition, Tweet Binder provided a full report with general metrics from all the tweets with #hikikomori in the defined period of time. This includes the total number of tweets and retweets, date and time tweeted, as well as (for this study) other secondary metrics: the temporal trend of the tweets, potential reach (the number of unique users that could have read the hashtag), and potential impact (the number of times that somebody could have read the hashtags).

**Figure 1:**
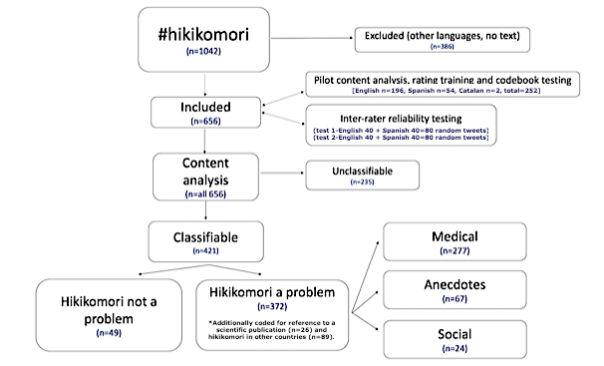
Flowchart of data management and content analysis.

Figure 1 shows a flowchart illustrating the process we followed for the analysis of the tweets, along with the number of tweets included and excluded.

### Content Analysis Process and Creation of the Codebook

All the retrieved tweets were directly inspected by 2 raters fluent in the included languages (VPS and MAM): both of them were psychiatric trainees and had previous experience in Twitter-related research, including the analysis of tweets. First, we scanned all of the tweets to classify them by language and excluded 386 of the total of 1042 tweets, according to our exclusion criteria. We created a codebook based on our research questions, previous experience analyzing tweets, and also determined by the most common themes we had observed reading the tweets. VPS and MAM analyzed 252 tweets separately to test the codebook. After an agreement on the codebook, the 2 raters classified, independently, a random set of 80 tweets (40 in English and 40 in Spanish), and the interrater reliability between both raters was assessed obtaining Kappa values ranging from 0.28 to 0.83 for the different categories and subcategories. Discrepancies were discussed between the raters and with the senior author (AT), and after revising the codebook, the interrater reliability was reassessed with a different set of 80 randomly selected tweets (40 in English and 40 in Spanish). As this resulted in adequate Kappa values ranging from 0.71 to 1.00 [45], the raters then proceeded to code the remaining tweets. For the remaining tweets, one of the raters (VPS) coded the tweets in Spanish, Italian, Catalan, and French, whereas the other rater (MAM) coded the tweets in English. One rater manually compiled the count of hashtags other than #hikikomori associated with the tweets.

**Table 1:** Category and subcategory definitions and examples of classification. Links and usernames within the tweets have been removed (usernames and personal names were replaced by XXXX). To seek for clarity, all the tweets reported here are in English (when tweets in other languages are reported, an English translation prepared by us is included in parentheses).

Category (or subcategory)		Definition	Examples of tweets
Unclassifiable		Not enough information, only links, spam, or random content.	“Furries is Love Furries is Life #Furries #Hikikomori”; “damn... that’s why I play this game again... kawaii character :D; #WhoCares #Lunatic #Hikikomori”
Hikikomori not as a problem		Positive or indifferent thoughts, attitudes, or behaviors related to hikikomori.	“I'm a hikikomori but I think outside the room. But many extroverts think like birds in a cage. #hikikomori”; “About to go full #Hikikomori. No regrets”
**Hikikomori as a problem**			
	Medical	Medical publications or reports, or events, campaigns, or interventions that present data or information related to hikikomori.	“Can we use #SocialMedia to identify socially withdrawn youth in China? Our latest paper on #hikikomori now out in @XXXX”; “#japan, Doctors began to observe it in the mid-1980s, with young men suffering from lethargy and refusing to communicate -- #hikikomori an insight #psychological ailment”.
	Anecdotes	Personal stories, testimonials, or third-person reports of people with hikikomori or related behaviors.	“#Hikikomori literally means ‘withdrawal from others’ in Japanese—follow the stories of a family affected by this modern-age social phenomenon as one day Nils decides to hide in his room and never leave. #Week53”.
	Social	Socially oriented issues related to hikikomori, including antistigma events, provision of social support, or related activities.	“If you are experiencing #hikikomori chat to others on #joinin247 #london #tokyo #osaka #kyoto #isolation #youarenotalone #endthesilence”
	Scientific reference	Explicit reference to a scientific publication (paper, presentation) in tweets with hikikomori as a problem.	“Can we use #SocialMedia to identify socially withdrawn youth in China? Our latest paper on #hikikomori now out in @XXXX”; “Secure Base Script and Psychological Dysfunction in Japanese Young Adults (Umemura et al 2018) #hikikomori via @XXXX”
	Other country	Explicit reference to hikikomori as a problem in a country other than Japan.	“#Hikikomori, è boom anche in Italia: migliaia di giovani si auto-recludono in casa”. (“#Hikikomori is also a boom in Italy: thousands of youth are self-reclusive at home”); “Can we use #SocialMedia to identify socially withdrawn youth in China? Our latest paper on #hikikomori now out in @XXXX”.

Table 1 illustrates the final content classification (codebook), providing the definitions and some examples of tweets coded in each category and subcategory. Regarding the tweet texts in the included languages, the first distinction was between *unclassifiable* and *classifiable* tweets. *Unclassifiable* tweets were no further analyzed, whereas the *classifiable* were then split between those referencing hikikomori *not as a problem* and those referring to hikikomori *as a problem*. The latter were subsequently coded in the subcategories of *medical*, *anecdotes*, and *social*. Finally, all the classifiable tweets were assessed to identify explicit references to scientific contents and reports to the existence of hikikomori or related behaviors in countries other than Japan (in that case, we recorded the countries explicitly referenced). In case of finding contents repeated exactly or almost identically in different tweets, they were classified in the same way as the first tweet encountered.

All the tweets were statistically analyzed to describe the number of tweets, retweets, and likes per language and category (and subcategory), considering retweets and likes as indices for reflecting the users’ interests in particular topics. We had previously reported the value of retweets in this regard [28], so we further calculated the Spearman correlation between retweets and likes for all the tweets to assess whether the likes could provide similar information. These analyses were conducted with the software packages STATA v14 (StataCorp) and SPSS Statistics v23.0.0.0 (IBM Corp).

This study received the approval of the University of Navarra Research Ethics Committee (October 11, 2018, modified on December 13, 2018) and is compliant with the research ethics principles of the Declaration of Helsinki (seventh revision, 2013). This study did not directly involve human subjects, nor did it include any intervention; instead uses only publicly available tweets (subject to universal access through the internet according to the Terms of Service that all users in Twitter accept). Nevertheless, we have taken care to not directly reveal in this report any username, and we have avoided citing tweets that could be offensive or compromised to someone.

## Results

Our search tool provided 1042 original tweets using #hikikomori in the established period, with 1433 retweets, a potential reach of 7,974,329, 10,613,856 potential impacts, and 908 contributors (ie, total number of different users posting with a given hashtag). As shown in Figure 1, our content analysis included 656 tweets (62.3% of the initial dataset). Of the total of 656 included tweets, 421 (64.8%) were considered classifiable. From these 421 classifiable tweets, 372 (88.36%) considered hikikomori as a problem.

**Figure 2:**
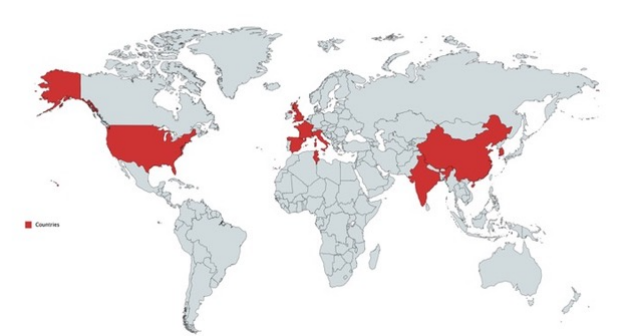
World map with countries where the presence of hikikomori was explicitly referenced by the tweets (Italy=63 tweets, United States=7 tweets, France=6 tweets, China=4 tweets, United Kingdom=3 tweets, and South Korea, India, Tunisia, and Spain=1 tweet each).

Table 2 shows the numbers and percentages of tweets per language and (sub)category. The distribution of tweets in each category was significantly different (*P*<.001) among languages. English and Italian were the most used languages (76.36%). The proportion of tweets referring to hikikomori as a nonproblematic behavior or lifestyle was highest in Spanish/Catalan tweets and lowest in Italian tweets. Italian was the language with the highest percentage of tweets considering hikikomori as a problem. Among tweets considering hikikomori as a problem, the medical contents were higher in all the languages in comparison with contents related to anecdotes or social. The language with the highest proportion of medical content was Italian, anecdotes was English, and social content was Italian. The proportion of tweets with explicit scientific references was very low in all languages; the highest proportion was found in English tweets. Only a minority of the tweets mentioned hikikomori as a problem outside Japan in all the languages except for Italian, where almost a third of the tweets had this reference. Figure 2 shows a world map including the countries other than Japan where hikikomori was explicitly reported as a problem.

The probability of retweet and like per category and subcategory is presented in Table 3. Tweets with an explicit reference to hikikomori outside Japan were statistically more retweeted and liked than those without that reference, whereas tweets with explicit scientific references were statistically more retweeted (but not more liked) than those tweets without that reference. 

**Table 2:** Descriptive characteristics of the original tweets included in the analysis, categorized by total amount per language and category. For each language and category or subcategory, total number of tweets (n) and relative proportions (%) are provided. In the first row, percentages of the total tweets in each language is calculated over the total of included tweets (ie, percentage of tweets in a given language among the total number of included tweets, 656); in the following rows, percentages are calculated for each category over the figures in the first row and in the same column (ie, percentage of tweets from a category among the total tweets in the given language or percentage of total tweets in each category among the total number of included tweets). Percentages are rounded to two decimals.

Category		English, n (%)	Italian, n (%)	Spanish and Catalan, n (%)	French, n (%)	Total, n (%)
Total tweets		290 (44.2)	211 (32.16)	61 (9.29)	94 (14.32)	656 (100)
Unclassifiable		125 (43.1)	54 (25.56)	19 (31.14)	37 (39.36)	235 (35.82)
Classifiable		165 (56.89)	157 (74.44)	42 (68.86)	57 (60.64)	421 (64.18)
Not a problem		30 (10.34)	0 (0)	13 (21.31)	6 (6.38)	49 (7.46)
**Problem**						
	Any	132 (45.51)	156 (73.93)	29 (47.54)	51 (54.25)	372 (56.7)
	Medical	79 (27.24)	132 (62.56)	19 (31.14)	47 (50)	277 (42.22)
	Anecdotes	43 (14.83)	13 (6.16)	8 (13.11)	3 (3.19)	67 (10.21)
	Social	10 (3.45)	11 (5.21)	2 (3.28)	1 (1.06)	24 (3.66)
	Scientific reference	18 (6.21)	5 (2.37)	1 (1.64)	2 (2.13)	26 (3.96)
	Other country	16 (5.18)	64 (30.33)	2 (3.28)	7 (7.45)	89 (13.57)

**Table 3:** Retweet to tweet ratio per category and subcategory.

Category and subcategory		Tweets, N		Retweets									Likes						
				N		Mean (SD)		Median		*P* value		N		Mean (SD)		Median		*P* value	
**Problem**										**.23**								**.82**	
	No		49		140		2.86 (11.00)		1				171		3.49 (16.54)		0		
	Yes		372		663		1.78 (2.66)		1				507		1.36 (3.84)		0		
**As a problem**										**.51**								**.65**	
	Medical		281		518		1.84 (2.82)		1				379		1.35 (4.11)		0		
	Anecdotes		67		109		1.63 (2.38)		1				110		1.64 (3.25)		0		
	Social		24		36		1.5 (1.10)		1				18		0.75 (0.90)		0.5		
**Scientific reference**										***.01***								**.10**	
	No		346		595		1.72 (2.67)		1				413		1.19 (3.21)		0		
	Yes		26		67		2.58 (2.56)		1.5				90		3.46 (8.46)		1		
**Reference to another country**										***.02***								***.02***	
	No		283		476		1.68 (2.81)		1				330		1.17 (3.41)		0		
	Yes		91		187		2.05 (2.11)		1				174		1.91 (4.89)		1		

^a^Number of retweets and likes per category and subcategory of all tweets in the included languages (here not separated by language), along with its mean (SD) and median values. Mann-Whitney *U* and Kruskall-Wallis tests were conducted to assess for statistical differences for the codification of each category. Statistical significance was considered when *P*<.05, and significant values are italicized.

**Figure 3:**
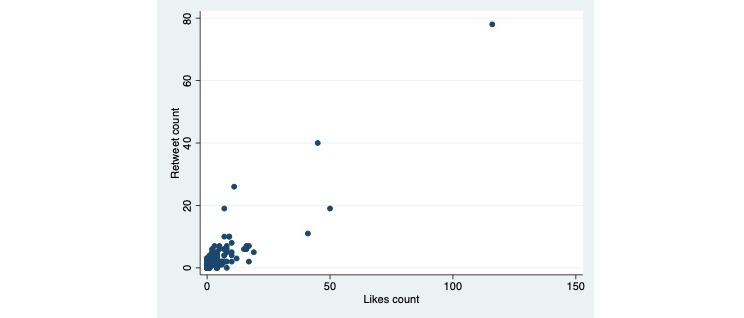
Correlation between retweets and likes among all the tweets in the sample (1042), showing a moderate (Spearman's rho: 0.54) and significant (*P*<.001) correlation between the 2 indices.

There were no differences in retweets and likes ratios among the different subcategories that considered hikikomori as a problem. The distribution of retweets per language followed an asymmetric distribution, with some individual tweets receiving a large number of retweets and likes; therefore, a statistical comparison of tweets and likes per language would be unreliable. Interestingly, accounting for all the tweets in the sample, and a secondary research result, the indices of retweet and like showed a moderate (Spearman’s rho: 0.54) and significant (*P*<.001) correlation (Figure 3).

Table 4 includes the *top 5* most frequently associated hashtags per language group. Looking at all languages, the most repeated words include references to Japan, the youth, and mental health or psychology.

The number of times each hashtag appears is shown in parentheses. Less than 5 are reported if the total number of associated hashtags per language was lesser than that number. More than 5 are reported when a tie occurred. For hashtags in languages other than English, an English translation is provided in parentheses. An interpretation of the hashtags is provided in the Discussion section.

**Figure 4:**
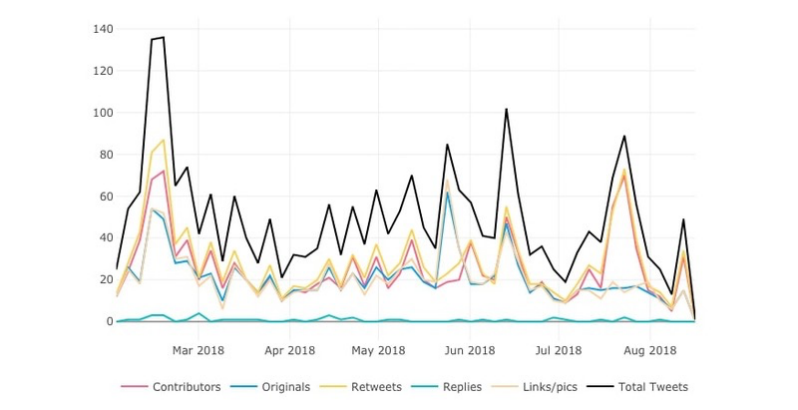
Time trend of all the tweets with #hikikomori in the period of study. The graph includes the number of contributors (different Twitter users publishing with this hashtag), original tweets, retweets, replies (tweets published as replies to the tweets with the hashtag), links and pictures included, and total number of tweets (original+retweets).

Finally, Figure 4 depicts the graphical timeline of tweets during the time frame selected, also as a secondary analysis. No temporal time trend pattern can be clearly identified.

**Table 4:** Top 5 hashtags associated with classifiable #hikikomori tweets, according to the language of the tweet. The values in brackets represent the number of tweets in the sample with the corresponding hashtags.

Language	*Top*^a^ related hashtags
English	#japan (32), #neet (10), #culture (9), #otaku (7), #mentalhealth (7)
Italian	#giovani (youth) (24), #italia (Italy) (16), giappone (Japan) (9), #isolamento (isolation) (8), #adolescenti (adolescents), #asocialita (asociality), #stareindisparte (being apart) and #autorrecludono (self-reclusion) (5)
Spanish	#japon (Japan) (5), #psicologia (psychology), #neetlife (4), #depresion (depression), #videojuegos (videogames), #anime, #adiccionalosvideojuegos (addiction to the videogames) (2)
French	#societe (society) (4), #sante (health) (2)

^a^*Top* hashtags refer to the most prevalent hashtags (different from #hikikomori) included in the tweets of the study.

## Discussion

### Comments on the Results

In this study, we investigated the tweets generated about hikikomori in several Indo-European languages (English, Italian, Spanish, Catalan, and French) which are mainly spoken in Western countries and altogether account for more than a billion native speakers. Our analysis strategy with Twitter Firehose data stream allows access to 100% of all public tweets within the search limits of words and time [44]. Thus, the conclusions were obtained from the results measured in the total population of tweets in those languages between Februray and mid-August 2018, and were not deduced from the analysis of a reduced sample.

To our best knowledge, this is the first study related to hikikomori in Twitter and the first to apply a mixed quantitative-qualitative approach to analyze tweets in different languages. English and Italian were the most used languages among those analyzed. A fair amount of the tweets could not be analyzed, which might be due to lack of information or context, or a random or nonsense use of the word hikikomori (in several cases, probably, as spam). Among the classified tweets, a minority described a perception of hikikomori or related behaviors as nonproblematic, perhaps describing a self-imposed lifestyle. Conversely, the majority of the tweets reported hikikomori as a problem, mainly in general terms (as an alarming social phenomenon or as a psychopathology), and at a lesser extent, some included first- or third-person testimonials, whereas the fewer were related to solidarity/activism. Explicit scientific references were barely found, but more than one-tenth of the tweets reported hikikomori in countries other than Japan, with a striking majority referring to Italy. Among the classifiable tweets, top associated hashtags were mainly related to Japan, the youth, the mental health psychology, and the society and culture. The time trend did not show markedly differentiated peaks of activity related to the hashtag.

Recent publications have proven the role of social media as a target for medical research and interventions [46,47]. Mental health disorders and conditions are topics of increased interest in Twitter [28], and this platform could be an enormous niche where many young people and socially withdrawn individuals could be reached. Our study tried to identify users reporting hikikomori-like symptoms or behaviors in themselves or others, in the search of potential targets for social media–based interventions. We did find tweets reporting these symptoms and behaviors in the categories of *not a problem* and *as a problem, anecdotes*. The similar number of tweets found in both categories (combined, up to 18% of the included tweets) shows that many users disclosing these symptoms or behaviors do not perceive them as a problem: this fact might be related with the common perspective of hikikomori as a self-imposed nonpathological lifestyle [25]. It furthermore provides an insight of the potential value of Twitter as an arena where affected individuals seek to revendicate their way of living or seeking for help.

In addition, Twitter might be considered as an indicator of real-time public opinion [46,48], as a source of general and medical information, and a framework for peer support through an online social community [49]. In this context, we thought that a mixed quantitative and qualitative analysis of tweets in Western languages related to hikikomori would be able to explore the public perceptions regarding this condition and the extent of this phenomenon within a global perspective. Our results suggest that hikikomori is not univocally perceived by users, probably existing a wide confusion in how it is widely understood. They also reflect that, whereas it is often perceived as a problem related to Japan, it might exist in other countries in the world, with particularly alarming references to Italy.

It should be noted that, currently, the literature related to medical research in hikikomori is also heterogenous and the concept has not yet reached an international consensus regarding its nature as a social phenomenon, a cultural-bond syndrome, or a psychopathological symptom or disorder per se [4]. Despite the increased evidence of its existence in countries other than Japan, especially in East Asia and in the West [1], and its frequent identification in mental health settings, the conceptual diversity, ignorance, and confusion in relation to how this problem is perceived in the psychiatric community seem high and should be addressed with cross-cultural international research.

Although few tweets included personal testimonies, their existence proves the value of Twitter as a means of communicating this kind of contents to a large public in spite of the still present public and self-stigma toward psychiatric conditions [50]. However, as Twitter allows for anonymity, it might be preferred by people with real or perceived personal or social restrictions, what makes this social network an ideal arena to discuss this topic. The lack of tweets with explicit scientific contents, which is probably, in part, related to our unavailability to explore the links attached, contrasted with its statistically significative higher chance to be retweeted (but not liked). These results are a stimulus to encourage researchers, editorials, and mass media outlets to explicitly highlight the results of scientific works in the social media, thus both offering evidence-based information to the general public and increasing the academic impact (ie, increasing the chance of citation) of the tweeted papers [51].

The global extension of the hikikomori phenomenon was confirmed in our analysis, as this term was used to name a problem existing in some countries across the world, and, despite the relatively low amount of tweets with these contents, they elicited a significant interest among users, as reflected by their statistically higher chance to be retweeted and liked. The increased interest in the emergence of hikikomori in countries other than Japan (especially in Italy, with some tweets reporting very high figures of prevalence in that country) reflected in retweets and likes of these contents contrasts with the still scarce published scientific literature of hikikomori and the lack of epidemiological figures and intervention guidelines in non-Japanese countries. Twitter is a fast social media reflecting real-time events and opinions; so, despite the heterogeneity and unnacuracy of many contents, clinicians, researchers, and policymakers should take them into account to address the problems and worries reflected in this social media and to rapidly detect and intervene on various health conditions [39].

We could not futher characterize the contents of the tweets addressing hikikomori as a medical problem, but they included news, epidemiological figures, opinions, research reports, and interventions. It would be valuable to explore the medical contents more in detail and compare them with the Twitter research in other health conditions. In addition, hikikomori’s core symptoms make social media a valuable tool to reach these patients [27]. As it has been shown that interventions in Twitter can modify health-related behavior [52], this platform could be a good scenario to promote a healthy lifestyle among at-risk individuals and encourage hikikomori patients to get in touch with offline health providers.

In accordance with previous research in Twitter, we considered retweets as a measure of users’ particular interest in a topic, which might be associated to the emotions elicited by the tweets in them [53]. In addition, we have explored the value of likes for the same purpose and found a moderate positive correlation between both indices. Further research could assess if retweets and likes are interchangeable metrics of interest or present different particularities between them.

### Limitations and Strengths

Some limitations should be noted in this study and need to be discussed in the light of its strengths. Most importantly, the codebook design and text analysis by 2 raters (with a third author as a supervisor), altogether with the mixed nature of this study (with a qualitative plus a quantitative approach), imply a degree of subjectivity, disagreement, and human error and constitute a challenge for its potential reproducibility by other authors. To address this issue, the study comprised a series of steps of initial review, design, and test of pilot codebooks and measurements of the interrater reliability. Thus, the process has been consistent, and the final ratings were expected to be reliable between the 2 raters. Consequently, in our opinion, this methodology is consistent with previous medical research studies in Twitter [54,55] and could be applied to different topics and by different authors. Although computerized machine-learning methods have been tested to automatically identify and classify topics in medical research in social media [56], we counted on the clinical expertise of the raters in Mental Health, which constitutes a qualitative advantage in relation to these automatized strategies.

To note, #hikikomori is not probably the unique word used in Twitter to reference this phenomenon (particularly outside Japan, where this concept may be unknown and where other words such as social isolation and withdrawal might be preferred), and tweets in Japanese (where one would expect to find the majority of the contents related to hikikomori) were not considered in this study and thus remain as a source of future cross-cultural research. However, although hikikomori is used as a limited term outside Japan (people might use other words to name it), this concept could also be a potential self-identification term for people who suffer from this problem and have no other words to name it [4].

The time frame was somewhat arbitrary but was selected to expect a reasonable number of tweets to classify. Apparently, the time trends of the retrieved tweets did not suggest the existence of a clear temporal pattern. A time trend was described in this study to rule out the presence of evident peaks in the activity of Twitter users regarding this topic, which has been observed in other medical conditions such as breast cancer during the so-called *awareness months* [57]. Many tweets were initially discarded owing to its language, according to the possibilities of the raters, so we could not analyze the contents of many tweets. Finally, the content analysis of tweets found the expected challenge of retrieving enough information to classify them. As tweets are limited in words and require no standard of linguistic or content correction to be published by anyone, and as we decided to avoid using information not included in the text itself (such as links or pictures, to limit the subjectivity in the rating, and for the sake of analytic efficiency), many tweets were rated as unclassifiable because of lacking enough information. Finally, the relatively small sample of tweets considered for content analysis precluded the possibility to separate independent samples for pilot testing of codebooks and interrater reliability, so the final classification might be somewhat biased. Nevertheless, the consistency of our method and the wide variety of tweets make us confident of having conducted a valuable mixed quantitative-qualitative exploration of this topic in a social media global context.

### Conclusions

In conclusion, hikikomori is a repeated word in different Western languages in Twitter, and despite its frequent use for uncertain or nonsense purposes, it is markedly perceived as a problem with strong associations with Japan, the society, the youth, and isolation. In addition, Twitter is a means to report personal stories, scientific publications, and the presence of this problem in non-Japanese societies. Our results provide a framework to take advantage of Twitter to provide users with accurate information, fight stigma, and reach a target population which might be unlikely to leave their rooms to ask for help by themselves. These kinds of interventions, using other social media platforms, have started to prove effective in people with hikikomori [27] and other potentially margined populations such as the military veterans [41]. Further research should take a cross-cultural perspective looking at tweets and other kinds of social media contents in Japanese and other Eastern and Western languages and test the potential of Web-based interventions to reach individuals with hikikomori or related behaviors and offer them support.

## Abbreviations

API: application programming interface
NEET: not in employment, education nor training
